# Influence of Implant Length on the Accuracy of Implant Placement in Sleeveless Guided Surgery: A Clinical Digital Superimposition Study

**DOI:** 10.7759/cureus.106790

**Published:** 2026-04-10

**Authors:** Charbel Bouyounes, Nabil Ghosn, Pierre Lahoud, Joseph Bassil

**Affiliations:** 1 Faculty of Dentistry, Université Saint-Joseph de Beyrouth, Beirut, LBN; 2 Department of Oral Surgery and Implantology, Université Saint-Joseph de Beyrouth, Beirut, LBN

**Keywords:** accuracy, computer-guided implant surgery, dental implants, digital superimposition, implant length, sleeveless surgical guide

## Abstract

Background and aim

Computer-guided implant surgery has improved implant placement accuracy by enabling precise transfer of the virtual plan to the surgical site. However, several factors may influence this accuracy, including surgical workflow, guide design, and implant-related variables. Among these, the effect of implant length remains insufficiently investigated. Therefore, this study aimed to evaluate the influence of implant length on the accuracy of implant placement using a sleeveless guided surgery protocol.

Materials and methods

Fifteen patients receiving implant-supported rehabilitation were included, with a total of 49 implants placed using a sleeveless guided surgery workflow (R2Gate SA, Megagen, Daegu, South Korea). Preoperative planning combined cone-beam CT and intraoral scan data. Placement accuracy was evaluated by digital superimposition of the planned and postoperative implant positions using scan body registration. Angular (°), coronal (mm), and apical (mm) deviations were measured. Implant length was analyzed as a continuous predictor using linear mixed-effects models, with patient as a random intercept.

Results

Increasing implant length was significantly associated with reduced angular deviation (β = -0.552° per 1 mm; 95% CI: -0.941 to -0.163; p = 0.0066) and reduced apical deviation (β = -0.108 mm per 1 mm; 95% CI: -0.205 to -0.011; p = 0.0305). In contrast, the association between implant length and coronal deviation did not reach statistical significance (β = -0.055 mm per 1 mm; 95% CI: -0.116 to 0.005; p = 0.0732).

Conclusions

Within the limitations of this study, implant length appeared to influence the accuracy of implant placement using sleeveless guided surgery. Longer implants were associated with reduced angular and apical deviations, suggesting improved placement accuracy. Further studies with larger samples are needed to confirm these findings and to better understand the role of implant-related factors in guided implant surgery accuracy.

## Introduction

Oral disorders are still among the most common health issues impacting people worldwide, resulting in higher healthcare and financial costs as well as a decline in quality of life [1]. Tooth loss indicates the history of oral and dental diseases across a person’s lifetime and is a measure of the oral health status of a community [2]. One of the most popular treatment choices in dentistry nowadays is dental implantology [3]. Immediate implantation has an extremely good survival rate; systematic review evidence indicates that immediate implant placement is associated with high survival, with a reported two-year survival rate of 98.4% in prospective studies [4].

In the late 1900s, guided planning and surgery were introduced [5,6]. Static computer-aided implant surgery (S-CAIS) is now a reliable and widely available therapeutic approach [7]. Prior studies have shown that freehand implant placement deviates more than procedures carried out with surgical guidance [8]. Increased prosthetic outcomes, including improved function, aesthetics, occlusion, and ideal load distribution, are made possible by precise implant placement [9]. Furthermore, developing prosthetic designs that support long-term maintenance and enable appropriate oral hygiene access requires precise implant placement [10].

The surgical guide’s guided drills control the implant osteotomy’s depth and angulation [11]. S-CAIS also has a number of practical restrictions and contraindications; its use may be problematic in several clinical circumstances, especially in patients with low interocclusal space, severe gag reflexes, or limited mouth opening, when simultaneous insertion of the surgical guide, drill, and handpiece becomes challenging or impossible. Cases with small edentulous spaces (less than 5 mm, which corresponds to the standard sleeve diameter) or circumstances where particularly long implants (14 mm or more) are anticipated are examples of further limitations. The design of implant surgical guides depends on preoperative data collection, which takes into account intraoral scans (obtained either by direct intraoral scanning or extraoral scanning of impressions) and cone-beam CT (CBCT) data [12].

The precision of guided implant placement can be assessed using a variety of techniques. Implant length has been suggested as a potential variable affecting the deviation between the virtually planned implant position and the final postoperative implant placement [13]. Implant position following surgery can be determined via impression coping or CBCT [14] or scan bodies connected to the placed implant fixture [15]. While accuracy relates to the variation shown in repeated measurements, trueness represents the degree of difference between the preoperative implant plan and the actual postoperative location [16].

The aim of the present study was to assess the accuracy of implant placement performed with a sleeveless guided surgery protocol using R2Gate SA software (Megagen, Daegu, South Korea), determining whether implant length influences the deviation between planned and actual implant positions.

## Materials and methods

Study setting and period

This study was conducted between April 21, 2025 and February 19, 2026 at the Department of Oral Surgery, Université Saint-Joseph de Beyrouth, Beirut, Lebanon.

Subjects

Fifteen patients meeting the inclusion criteria were enrolled in the present study, requiring the placement of a total of 49 dental implants. Inclusion criteria included age greater than 20 years, satisfactory oral hygiene, absence of acute infection at the surgical site, absence of periodontal pockets exceeding 3 mm at the intended implant site, and absence of buccal bone wall defects such as dehiscence or fenestration. Patients presenting with systemic or bone-related conditions that could compromise peri-implant healing were excluded from the study.

Instrumentation and measurement

Implant placement accuracy was evaluated by comparing the preoperative virtual implant plan with the postoperative implant position using digital superimposition. Postoperative intraoral scans were obtained using an intraoral scanner (Medit i700, Medit Corp., Seoul, South Korea) with a scan body connected to the implant fixture.

The scan data were imported into the implant planning software (R2Gate, Megagen) and aligned with the preoperative planning dataset. Following superimposition, the actual implant position was reconstructed based on the scan body geometry. The planned and placed implant positions were then simultaneously visualized within the software environment. The following deviations between planned and actual implant positions were calculated using the measurement tools provided by the software: angular deviation (degrees), coronal deviation (mm), and apical deviation (mm).

Materials

Implant planning and guided surgery were performed using the R2Gate digital implant planning system (Megagen). Preoperative CBCT scans and intraoral surface scans were used for virtual implant planning. Sleeveless surgical guides were designed within the planning software and fabricated for guided implant placement. All implants were placed using the Megagen implant system (Megagen) according to the manufacturer’s recommended drilling protocol. Postoperative digital impressions were obtained using an intraoral scanner with a compatible scan body to capture the implant position for subsequent deviation analysis.

Procedures

Preoperative CBCT radiographs and intraoral digital scans were obtained for all patients. DICOM files from CBCT imaging were combined with STL files from intraoral scans, and implant planning was performed using 3D software (R2Gate SA, Megagen). Implants were placed according to a prosthetically driven approach after evaluation of bone volume and density, while considering adjacent anatomical structures such as the maxillary sinus and inferior alveolar nerve canal.

During planning, minimum safety distances were respected: 2 mm between implants and anatomical structures, 2 mm between anchor pins, 2 mm between anchor pins and implants, and 2 mm between anchor pins and anatomical structures. The finalized planning data were exported to the R2Gate SA™ software for surgical guide design, where the guide hole was automatically positioned according to the implant dimensions and the manufacturer’s surgical kit specifications.

**Figure 1:**
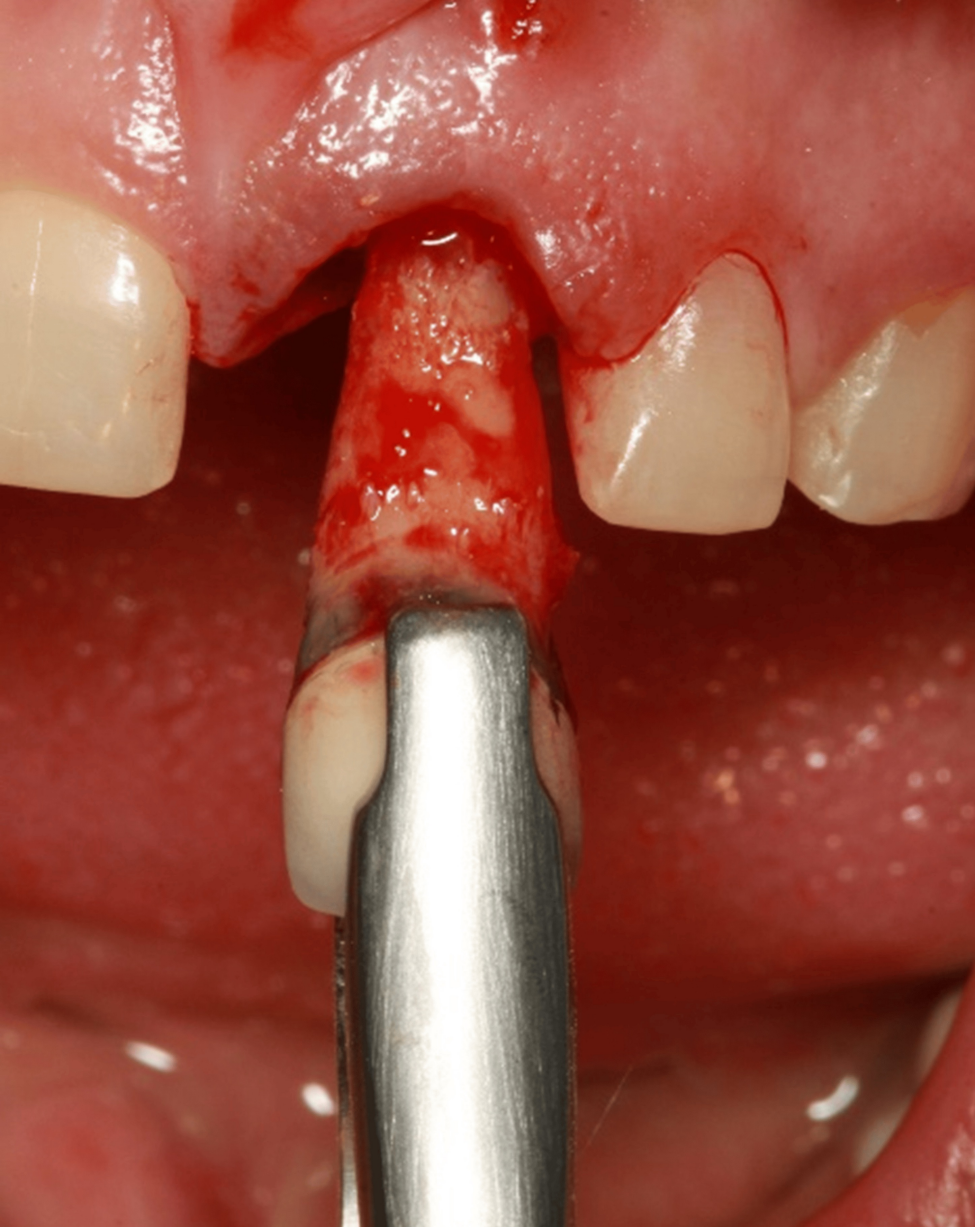
Atraumatic tooth extraction Atraumatic tooth extraction performed using specialized extraction instruments to preserve the integrity of the alveolar bone and surrounding soft tissue.

All procedures were performed by a single experienced operator to ensure consistency. When tooth extraction and immediate implant placement were indicated, atraumatic extraction was conducted (Figure 1). The surgical guide was then positioned and stabilized to ensure proper seating and stability.

**Figure 2:**
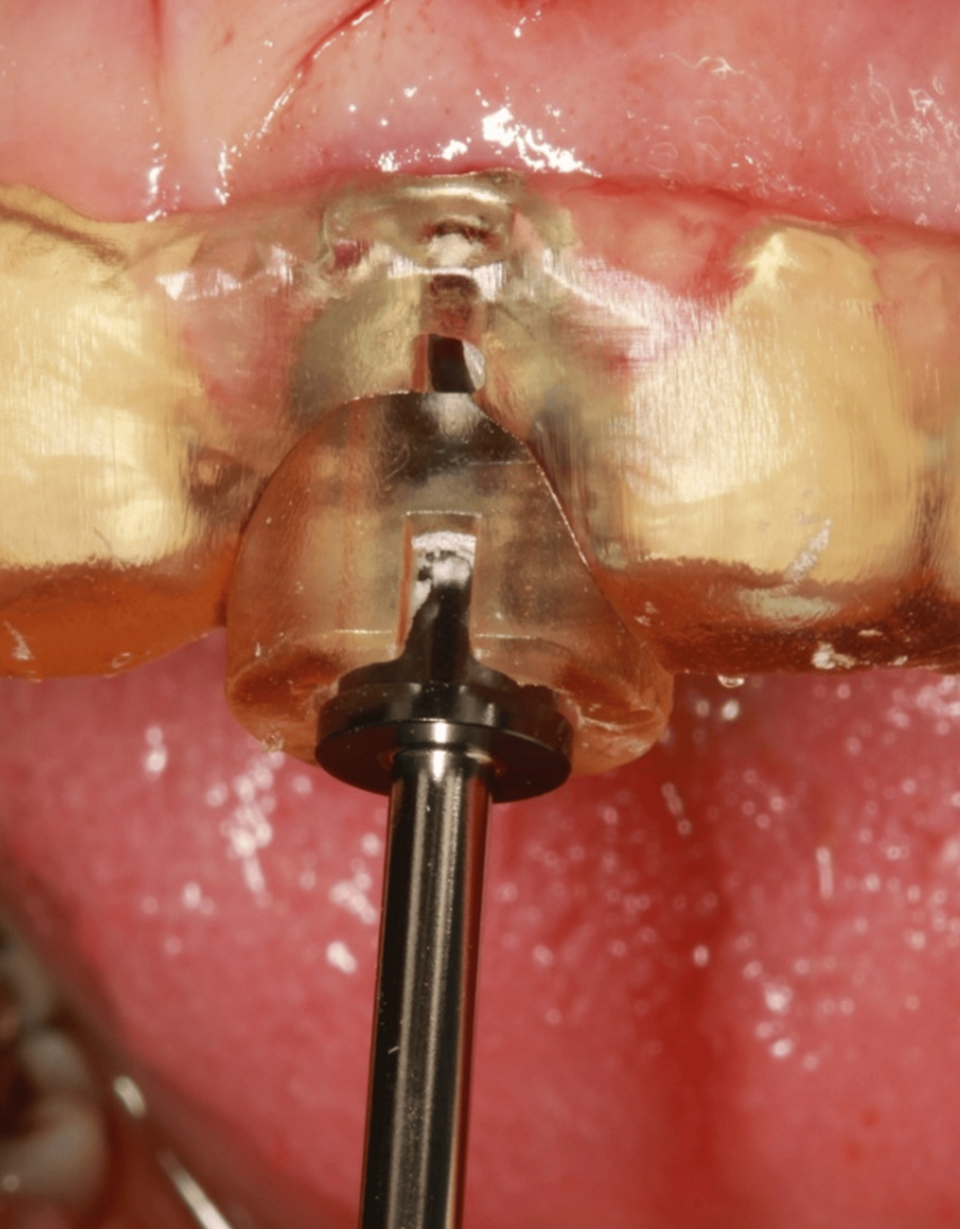
Guided drilling through a sleeveless surgical template Tooth-supported surgical guide in place during osteotomy preparation, demonstrating precise fit and stability while guiding the drill for accurate implant positioning.

For each procedure, the drilling sequence was selected based on the implant dimensions (Figure 2) according to the manufacturer’s recommendations using the R2Gate surgical kit. Implants were then inserted according to the preoperatively planned position. Implant primary stability was evaluated using resonance frequency analysis with an Ostell ISQ device (Integration Diagnostics AB, Göteborg, Sweden).

Accuracy assessment

Radiographic CBCT scans were obtained preoperatively (T0). Intraoral scans were taken both preoperatively and postoperatively with a scan body connected to the implant to allow digital registration (T0-T1). Each patient dataset was initially prepared using R2Gate SA software. Superimposition of preoperative and postoperative datasets was performed using the R2Aid function. For the alignment process, the postoperative scan and scan body file were imported into the initial planning file. The preoperative intraoral scan served as the reference model, while the postoperative scan was used as the target STL file.

Manual alignment was performed by selecting three corresponding reference points on both datasets. After alignment, the matched STL file was exported and saved. The implant planning file was then reopened, and the aligned STL file was imported into the STL Match Unit as a supporting model.

**Figure 3:**
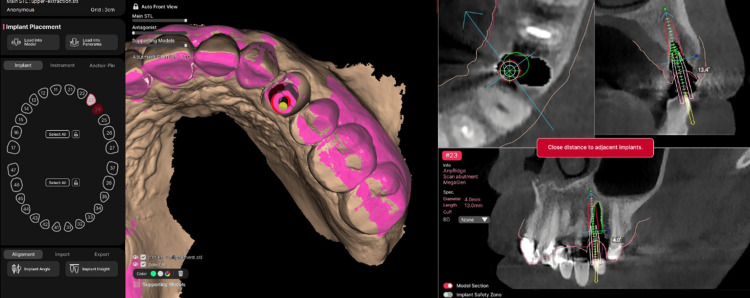
Alignment of the preoperative and postoperative datasets R2Gate SA software displaying preoperative and postoperative scans simultaneously, illustrating the alignment process using two scan bodies, one preoperative and one postoperative, prior to final superimposition.

**Figure 4:**
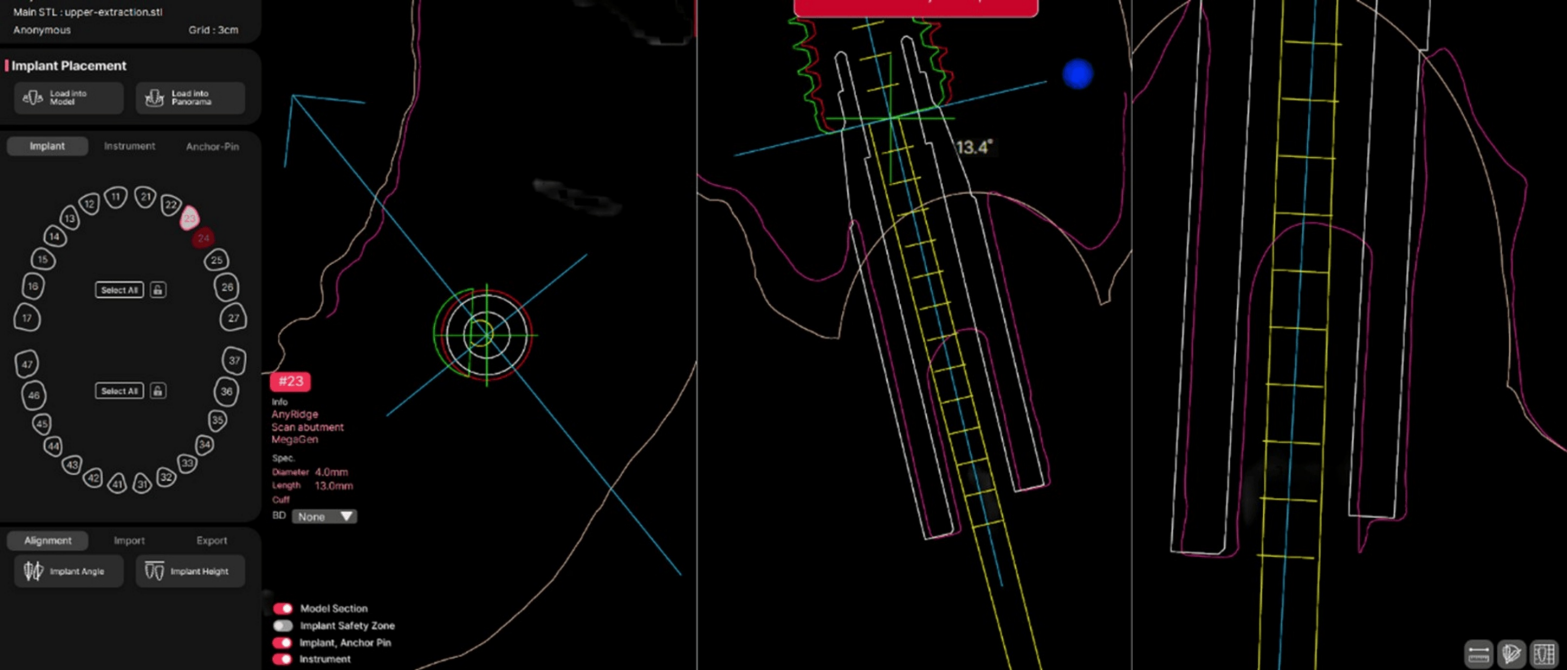
Reconstruction of the placed implant position based on the scan body Pre-alignment visualization of the preoperative and postoperative scan bodies displayed in three orthogonal 2D views (axial, sagittal, and coronal) prior to the alignment process.

A virtual implant was reconstructed based on the scan body reference to represent the actual implant position. The implant diameter and implant length were selected from the software implant library to match the implant placed clinically. Alignment was verified in both 3D and 2D views to ensure accurate positioning (Figure 3, Figure 4).

**Figure 5:**
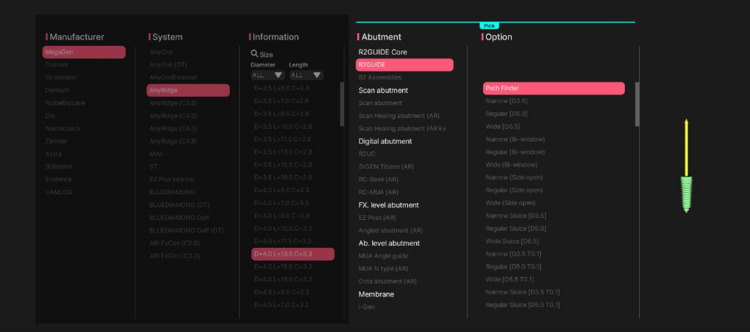
Selection of the Pathfinder abutment for the deviation assessment process Display of the R2Gate software library, highlighting the selection and use of the Pathfinder abutment to achieve precise alignment for deviation assessment.

After successful alignment, the planned and actual implant positions were displayed simultaneously. The Pathfinder tool was used as the abutment reference to determine the center of the implant platform and calculate positional deviations (Figure 5).

**Figure 6:**
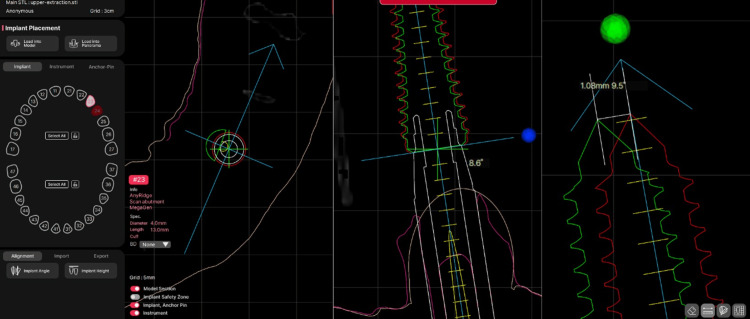
Measurement of apical deviation 2D views within R2Gate software demonstrating the use of measurement tools to assess the distance between planned and postoperative implant positions, illustrating apical deviation.

**Figure 7:**
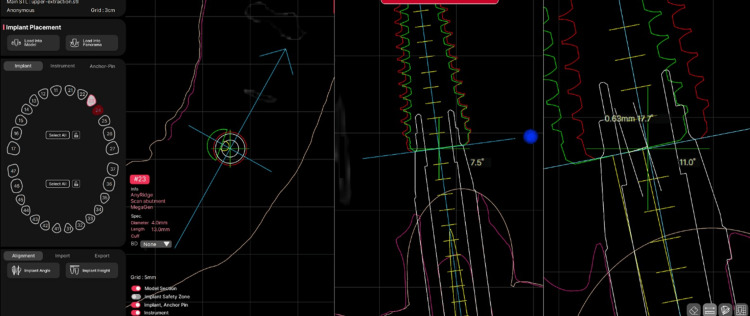
Measurement of coronal deviation 2D views within R2Gate software demonstrating the use of measurement tools to evaluate the distance between planned and postoperative implant positions at the coronal level, illustrating coronal deviation.

Coronal and apical deviations between the planned and actual implant positions were measured in millimeters using the R2Gate measurement tools (Figure 6, Figure 7).

**Figure 8:**
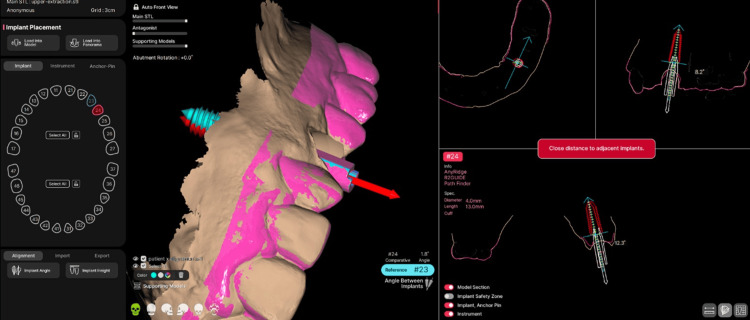
Measurement of angular deviation R2Gate software interface showing the automatic calculation of angular deviation between planned and postoperative implant axes using the angle deviation tool.

Angular deviation between the implant axes was calculated in degrees using the “Angle Between Implants” tool (Figure 8). All measurements were performed by a single operator to minimize measurement variability. For each implant, three outcome variables were obtained: angular deviation (degrees), coronal deviation (mm), and apical deviation (mm).

Statistical analysis

All statistical analyses were performed using RStudio (version 2025.09.2, RStudio Team, Boston, MA, USA). Descriptive statistics were reported as mean ± SD. To investigate the influence of implant length on placement accuracy, linear mixed-effects models were applied. Implant length (mm) was included as a continuous predictor variable, while patient identifier was entered as a random intercept to account for clustering of multiple implants within the same patient. Separate models were fitted for angular, coronal, and apical deviations. Statistical significance was set at p < 0.05.

## Results

Study population characteristics

The study included 15 patients with a total of 49 implants. Among the participants, seven were males, and eight were females. A total of 27 implants were placed in the maxilla, while the remaining 22 implants were placed in the mandible. Detailed demographic and clinical characteristics are presented in Table 1. Angular, coronal, and apical deviations were available for all implants. As multiple implants were placed in some patients, subsequent inferential analyses were structured to account for within-patient clustering.

**Table 1:** Demographic and clinical characteristics of the study population

Variable	Value
Number of patients	15
Number of implants	49
Sex	
Male	7 (46.7%)
Female	8 (53.3%)
Implant location	
Maxilla	27 (55.1%)
Mandible	22 (44.9%)

Descriptive analysis of implant placement accuracy

Angular, coronal, and apical deviations are reported as mean ± SD.

Effect of implant length on accuracy

Implant length was evaluated as a continuous predictor in linear mixed-effects models (random intercept for patient). Increasing implant length was associated with a statistically significant reduction in angular deviation (β = -0.552° per 1 mm, 95% CI: -0.941 to -0.163, p = 0.0066*) and a statistically significant reduction in apical deviation (β = -0.108 mm per 1 mm, 95% CI: -0.205 to -0.011, p = 0.0305*). In contrast, the association between implant length and coronal deviation did not reach statistical significance (β = -0.055 mm per 1 mm, 95% CI: -0.116 to 0.005, p = 0.0732) (Table 2).

**Table 2:** Implant placement accuracy according to implant length * indicates statistically significant results (p < 0.05).

Outcome	β (per 1 mm)	95% CI	p-Value
Angular deviation (°)	-0.552	-0.941 to -0.163	0.0066*
Coronal deviation (mm)	-0.055	-0.116 to 0.005	0.0732
Apical deviation (mm)	-0.108	-0.205 to -0.011	0.0305*

Multivariable analysis of factors associated with placement accuracy

Multivariable linear mixed-effects models were used to identify factors independently associated with implant placement accuracy while accounting for clustering of multiple implants within the same patient. Separate models were fitted for angular, coronal, and apical deviations, with patient identifier included as a random intercept.

As shown in Table 3, implant length was significantly associated with all three accuracy outcomes. Specifically, increasing implant length was associated with a reduction in angular deviation (β = -0.797; 95% CI: -1.239 to -0.354; p < 0.001), coronal deviation (β = -0.069; 95% CI: -0.135 to -0.003; p = 0.039), and apical deviation (β = -0.157; 95% CI: -0.264 to -0.050; p = 0.004).

**Table 3:** Multivariable analysis of factors associated with implant placement accuracy

Outcome	Predictor	β (adjusted)	95% CI	p-Value
Angular deviation (°)	Implant length (per 1 mm)	-0.797	-1.239 to -0.354	<0.001
Coronal deviation (mm)	Implant length (per 1 mm)	-0.069	-0.135 to -0.003	0.039
Apical deviation (mm)	Implant length (per 1 mm)	-0.157	-0.264 to -0.050	0.004

## Discussion

With the advancement of computer-aided design/computer-aided manufacturing and AI technologies, the use of surgical guidance in clinical instances has grown recently [17]. Accurate preoperative planning is essential for effective implant placement [18]. This study evaluates the accuracy of implant placement performed using a sleeveless guided surgery workflow (Megagen), with particular focus on the effect of implant length on deviations between planned and actual implant positions. Previous studies have demonstrated that the accuracy of guided implant surgery may be influenced by several factors, including guide design, manufacturing methods, and surgical workflow [19,20].

The present study evaluated the influence of implant length on the accuracy of implant placement performed using a sleeveless guided surgery protocol. The findings demonstrated that increasing implant length was significantly associated with reduced angular and apical deviations between the planned and actual implant positions. In contrast, no statistically significant association was observed between implant length and coronal deviation.

Mohammed and Jalal [21] evaluated the combined influence of implant angulation and implant length on implant placement accuracy using tooth-supported fully guided surgical templates fabricated through the RealGUIDE digital workflow. Their results showed that implant angulation significantly influenced deviation patterns, with implants placed at 0° demonstrating the highest accuracy. In contrast, the present study assessed implant placement accuracy using a sleeveless guided surgery workflow, which eliminates the mechanical tolerance between the drill and the guide sleeve. This difference in guide design may partly explain the variations observed between studies. Additionally, methodological differences, including sample size and the use of CBCT-based superimposition, which may introduce registration errors, could also contribute to the observed discrepancies [22].

In our study, an intraoral scan was taken after surgery while the scan abutments were positioned on implants. This scan was then incorporated into the original CBCT-based planning to provide an additional alignment reference. Additionally, STL file superimposition is typically more reliable than DICOM file superimposition [23], avoiding volumetric artifacts and segmentation inaccuracies associated with CBCT datasets.

Furthermore, the present findings suggest that increasing implant length may contribute to improved angular and apical accuracy. One possible explanation is that longer implants require deeper osteotomy preparation, which may enhance stabilization of the drilling trajectory within the bone channel. As the drill advances through a longer osteotomy, the surrounding bone walls may help maintain the planned angulation and reduce lateral displacement, thereby minimizing positional deviations. In contrast, shorter implants involve shallower osteotomies, where the drill may be more susceptible to minor lateral movements, potentially increasing the risk of apical deviation. This biomechanical mechanism may partly explain the inverse relationship observed between implant length and deviation values in the present study.

In addition, strict adherence to the surgical protocol and increased operator experience may have contributed to minimizing technical and positional errors [24]. Although guided implant surgery is inherently associated with minor deviations, the reduced discrepancy observed in this study suggests that the applied workflow offers superior control over implant trajectory and depth.

To the authors’ knowledge, limited evidence exists regarding the influence of implant length on placement accuracy in sleeveless guided implant surgery systems. Most studies on guided implant accuracy have focused on factors such as guide design, support type, manufacturing methods, and surgical workflow, while implant-related variables, particularly implant length, have received less attention. Furthermore, existing investigations have mainly evaluated conventional sleeve-based systems, leaving the potential effect of implant length in sleeveless guided surgery largely unexplored. Therefore, the present study aims to evaluate the relationship between implant length and placement accuracy within a sleeveless guided surgical workflow.

This study presents several strengths. The use of scan bodies enhanced 3D superimposition and reduced alignment errors. In addition, all surgical planning and procedures were performed by a single operator, ensuring protocol standardization and minimizing technique-related variability. Moreover, superimposition and measurement procedures were carried out by an independent operator not involved in the surgical phase, reducing observer bias and improving the reliability of the accuracy assessment.

However, some limitations should be acknowledged. The retrospective design represents an important limitation, and the relatively small sample size of 15 patients may restrict the statistical generalizability of the results. In addition, the use of a single operator may limit the extrapolation of the findings to less experienced clinicians. Finally, this study focused on a single implant system (Megagen), which may limit the applicability of the results to other systems. Further clinical and experimental studies are required to confirm these findings and better understand the influence of implant length on placement accuracy.

## Conclusions

Within the limitations of the present study, implant length appeared to influence the accuracy of implant placement performed using a sleeveless guided surgery protocol. Increasing implant length was associated with significantly reduced angular and apical deviations between the planned and actual implant positions, while no significant association was observed with coronal deviation. These findings suggest that implant length may represent a contributing factor to placement accuracy in guided implant surgery. Further studies with larger sample sizes and different guided surgery systems are required to confirm these results and better understand the relationship between implant length and implant placement accuracy.
